# Circulating tumour DNA (ctDNA) as a biomarker in metachronous melanoma and colorectal cancer- a case report

**DOI:** 10.1186/s12885-019-6336-3

**Published:** 2019-11-14

**Authors:** Leslie Calapre, Lydia Warburton, Michael Millward, Elin S. Gray

**Affiliations:** 1 School of Medical Science, Edith Cowan University, Joondalup, WA Australia; 2 Department of Medical Oncology, Sir Charles Gairdner Hospital, Nedlands, WA Australia; 3 School of Medicine and Pharmacology, The University of Western Australia, Crawley, Western Australia Australia; 4 School of Biomedical Science, University of Western Australia, Crawley, WA Australia

**Keywords:** BRAF, Melanoma, Circulating tumor DNA, Colon cancer, Survivorship, Case report

## Abstract

**Background:**

Circulating tumour DNA (ctDNA) has emerged as a promising blood-based biomarker for monitoring disease status of patients with advanced cancers. The presence of ctDNA in the blood is a result of biological processes, namely tumour cell apoptosis and/or necrosis, and can be used to monitor different cancers by targeting cancer-specific mutation.

**Case presentation:**

We present the case of a 67 year old Caucasian male that was initially treated with BRAF inhibitors followed by anti-CTLA4 and then anti-PD1 immunotherapy for metastatic melanoma but later developed colorectal cancer. The kinetics of ctDNA derived from each cancer type were monitored targeting *BRAF V600R* (melanoma) and *KRAS G13D* (colon cancer), specifically reflected the status of the patient’s tumours. In fact, the discordant pattern of BRAF and KRAS ctDNA was significantly correlated with the clinical response of melanoma to pembrolizumab treatment and progression of colorectal cancer noted by PET and/or CT scan. Based on these results, ctDNA can be used to specifically clarify disease status of patients with metachronous cancers.

**Conclusions:**

Using cancer-specific mutational targets, we report here for the first time the efficacy of ctDNA to accurately provide a comprehensive outlook of the tumour status of two different cancers within one patient. Thus, ctDNA analysis has a potential clinical utility to delineate clinical information in patients with multiple cancer types.

## Background

In recent years, tumour-derived cell free DNA (ctDNA) has emerged as a promising biomarker of disease status for metastatic cancer [1–3]. Plasma ctDNA are short nucleic acid fragments (~ 166 bp) thought to be released in the systemic circulation as a result of tumour cell apoptosis and/or necrosis [4, 5]. Previous studies have shown that ctDNA carries genetic information from the entire tumour genome and can therefore provide insights into clonal heterogeneity and evolution of all solid cancers present at any one time [6, 7]. As analysis of ctDNA can be tailored for different cancers by targeting specific mutations, it provides detailed information via a minimally invasive ‘liquid biopsy’, eliminating the morbidity associated with serial sampling of tumours for monitoring patients with any advanced solid cancers.

Various studies in breast, lung and colorectal cancers have demonstrated the potential clinical application of ctDNA analysis at each stage of cancer management: early diagnosis [5, 8], molecular profiling [6, 9–11], prognostication [5, 12, 13], detection of residual disease [14, 15], monitoring response and clonal evolution [16–20]. In melanoma, several studies have also shown the efficacy of utilising ctDNA for monitoring patients with *BRAF* mutant tumours, particularly in the context of treatment response and identification of mechanisms of resistance to *BRAF* inhibitors [7, 21–26]. These studies provide credence to the utility of ctDNA for patient monitoring only in the context of singular cancer. To date, ctDNA remains unutilised in clinical management of patients with multiple tumour types and/or those metachronous cancers where new primary tumours arise that are unrelated to the original malignancy. In this case study, we demonstrated the efficiency of ctDNA to delineate the different status of both melanoma and colorectal cancers in a single patient.

## Case presentation

A 67-year old male was investigated in our institution in 2012 for weight loss and abdominal pain. He was otherwise fit and well, with no significant comorbid medical history. He was not on any regular medications, had no known allergies and had no significant family history. Computed tomography (CT) revealed moderate ascites and a large splenic mass. Fine needle splenic aspirate was non-diagnostic and therefore a therapeutic/diagnostic splenectomy was performed. Metastatic melanoma was confirmed histologically, and further testing confirmed a *BRAF V600R* mutation via Sanger sequencing. In July 2014, he commenced dabrafenib and trametinib treatment for progressive disease but suffered unacceptable toxicity, which led to the cessation of the combined targeted therapies.

**Fig. 1:**
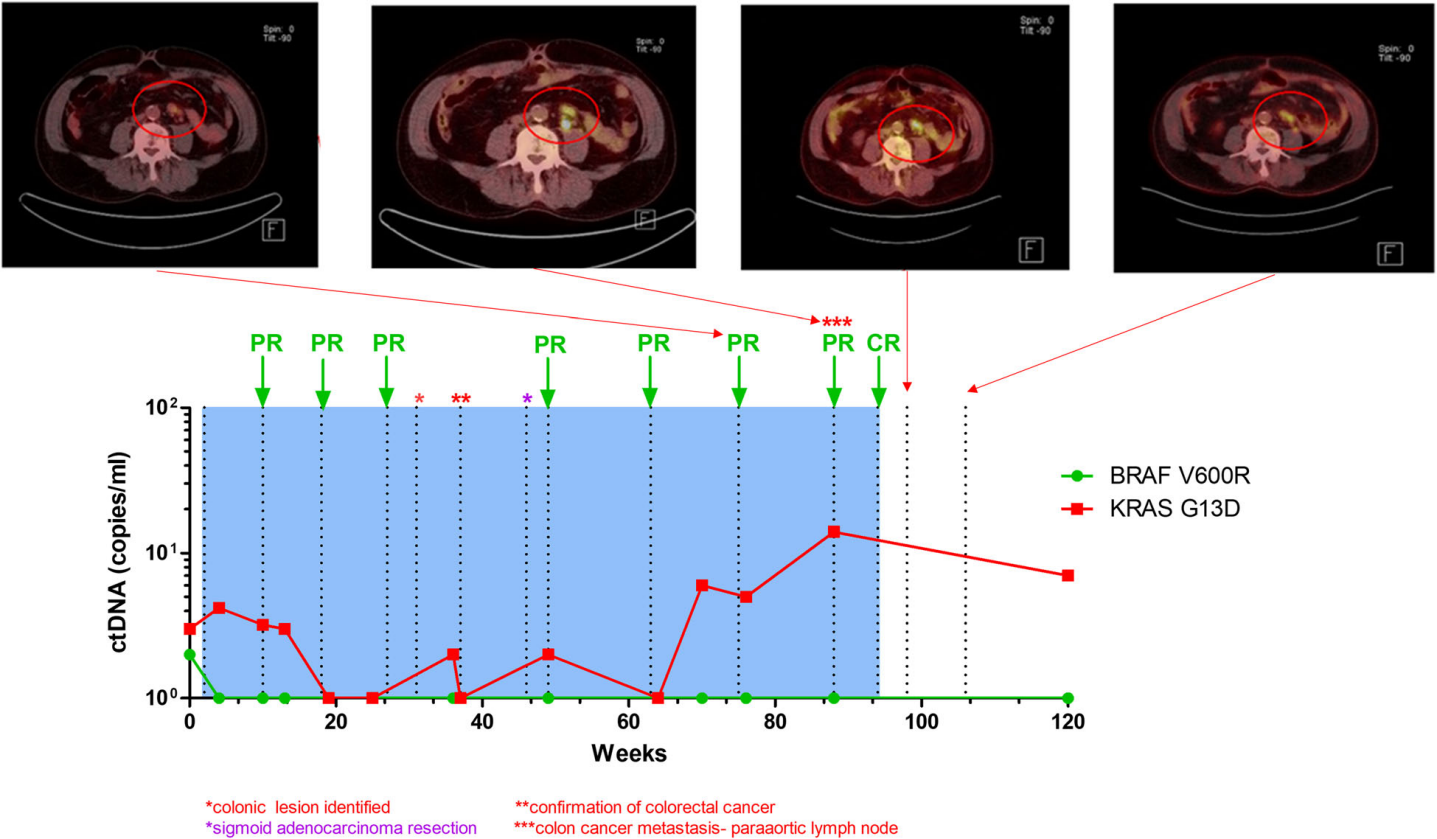
ctDNA analysis can discriminate the status of different tumours in a patient with both melanoma and colorectal cancer. Levels of *BRAF* and *KRAS* ctDNA (green) inform of the status of melanoma and colon cancer respectively. Clinical partial response (PR) and complete response (CR) annotations are indicative of melanoma response to pembrolizumab as measured by RECIST on CT imaging. PET scan images associated with four different timepoints with differential *BRAF* and *KRAS* ctDNA levels

At progression the patient was subsequently treated with four doses of ipilimumab (3 mg/kg three weekly) but was found to have disease progression on the first response assessment CT scan. Confirmed progression in lung metastases and the intra-abdominal nodal disease led to commencement of anti PD-1 therapy (pembrolizumab 2 mg/kg three weekly) in March 2015 (week 2, Fig. 1). He completed 28 cycles (week 94) of pembrolizumab and achieved a complete metabolic response on PET at six months in all the previously identified metastatic sites. He tolerated treatment well with vitiligo as the sole side effect.

However, PET at 32 weeks identified a new FDG avid lesion within the sigmoid colon. This was investigated with colonoscopy and tissue biopsy confirmed a low grade sigmoid adenocarcinoma. He proceeded to a subtotal colectomy, ilio-sigmoid anastomosis and lymph node dissection in January 2016 (week 46). Histopathology confirmed a stage III (T4N1M0 AJCC 7th edition) low grade sigmoid adenocarcinoma with 3/33 lymph nodes involved. The tumour had no mismatch repair deficiency. Molecular analysis using next generation sequencing via the Illumina Trusight tumour panel showed the primary tumour to be *KRAS p. G13D* mutant, *NRAS* and *BRAF* wild type. Post-operative CEA measurements were negative.

Adjuvant chemotherapy for colon cancer was offered but the patient decided to continue with pembrolizumab for metastatic melanoma and declined chemotherapy. In November 2016, eleven months after curative resection of primary colorectal cancer, para-aortic nodes enlarged marginally and became intensely FDG avid on PET despite on-going pembrolizumab. Biopsy of an enlarging para-aortic node at approximately week 88 confirmed metastatic colorectal cancer. Molecular analysis of the colorectal metastasis confirmed *KRAS p. G13D* mutation. It is of note that the patient had no other sites of disease progression and remained in complete response from metastatic melanoma, which led to cessation of pembrolizumab treatment.

The recurrence was unresectable and the patient was offered palliative FOLFOX chemotherapy with bevacizumab (B) but chose to undergo observation with three monthly clinical and radiological reviews. His imaging demonstrated RECIST (Response Evaluation Criteria in Solid Tumours) stable disease for 18 months and he then progressed with new liver and lung lesions. He has recently commenced B-FOLFOX chemotherapy with response assessment pending.

### ctDNA screening and monitoring

In parallel to the imaging scans, the patient was monitored for melanoma and colorectal cancer by tracking *BRAF p.V600R* and *KRAS p.G13D* mutations in ctDNA respectively. Blood samples were collected in EDTA and Streck tubes. Plasma was separated within 24 h by centrifugation at 300 g for 20 min, followed by a second centrifugation at 4700 g for 10 min, and then stored at -80 °C until extraction. Cell-free DNA (cfDNA) was extracted from 5 ml of plasma using the QIAamp Circulating Nucleic Acid Kit (Qiagen) as per the manufacturer’s instructions. Analysis of plasma ctDNA was carried out using an in-house BRAF p.V600R assays [27] and a commercial KRAS p.G13D (Bio-Rad) for droplet digital PCR (ddPCR). Protocols used for ddPCR analysis were as previously described [21, 28] and ctDNA levels were calculated based on the number of copies per millilitres of plasma (c/mL).

Plasma analysis demonstrated the presence of *BRAF V600R* ctDNA at baseline prior to initiating pembrolizumab, which became undetectable at subsequent follow-up (weeks 2–10.) The patient achieved sustained partial response to pembrolizumab (week 18–49) by CT and complete metabolic response by PET scan, which was supported by his corresponding ctDNA data (Fig. 1). As predicted, the patient’s blood sample at the time of colorectal cancer diagnosis (week 36) had detectable *KRAS* mutant ctDNA (2 c/mL). Retrospective analysis of the previous blood samples revealed detectable levels of *KRAS* mutant ctDNA prior to immunotherapy (3 c/mL), suggesting that colorectal cancer may have already been present at the time of stage IV melanoma diagnosis. Subsequent plasma samples (weeks 2–49) were also found to have detectable KRAS mutant ctDNA, albeit at consistently low levels that ranged from 2 to 4 c/ml, with the exception of the blood sample collected at week 64 that was negative for ctDNA. In this case, real time knowledge of detectable ctDNA following curative bowel resection, implying residual microscopic disease, and the negative *BRAF* mutant melanoma ctDNA may have influenced and ultimately changed the clinician and patients decision from not having adjuvant chemotherapy, to receiving it.

Increased *KRAS* mutant ctDNA was further observed at week 70 (6 c/mL), which provided an early indication of disease progression. Prior to the cessation of pembrolizumab corresponding to the melanoma complete response, *KRAS* mutant ctDNA levels was at its peak (14 c/mL). A final ctDNA assessment at week 119, revealed that *BRAF V600R* ctDNA continues to be undetectable which is consistent with sustained complete response of melanoma. Nevertheless, ctDNA for *KRAS G13D* remained high (7 c/ml) suggesting possible radiologically undetectable progression of the patient’s untreated colon cancer.

## Discussion and conclusion

This case study highlights the evolving role of ctDNA in detecting metachronous cancers. Development of new primary tumours that are unrelated to the original malignancy have become a significant adverse effect in patients with metastatic cancers who achieved sustained or durable disease control in response to targeted and/or systemic therapy. Thus, oncologists should be vigilant to the possibility that patients are at continued risk for new and separate malignancies.

Previous studies have demonstrated the clinical utility of ctDNA as a biomarker of disease status in patients with metastatic cancers, particularly in the context of singular tumour types. However, to date there has been no report of the clinical utility of ctDNA to delineate status of different tumour types within patients with multiple cancers. In this case study, we demonstrated the efficiency of ctDNA, by targeting tumour-specific mutations to specifically inform treatment response and tumour status in a patient with both melanoma and colorectal cancer. Given the increased risk of cancer patients to develop other malignant tumours, this study supports the potential clinical use of ctDNA for profiling of other emerging lesions and identification of their origin. Plasma ctDNA may be useful for accurate stratification of treatment response in patients with two or more different tumour types, providing better perspective of disease status for more informed treatment options. In this setting, pan-cancer ctDNA testing can aid on the early detection of metachronous cancers.

The low melanoma derived ctDNA at baseline may be the result of partial disease control by the previous ipilimumab therapy, although not evident by the CT scan performed. The immediate drop and undetectable levels of *BRAF* mutant ctDNA during pembrolizumab treatment indicated the response of the patient’s melanoma tumour to this treatment. On the other hand, colon cancer derived ctDNA (*KRAS*) was also detectable at baseline, and given that it is at similar concentrations as *BRAF*, the patient’s melanoma and colorectal tumour burden may be relatively similar. Studies have shown that ctDNA is readily detectable in early stages colon cancer patients [29, 30] which may explain the detectability of colon cancer ctDNA in this patient. Nevertheless, we like to note that we observed fluctuations of the level of *KRAS* mutant ctDNA at the time of pembrolizumab treatment. Interestingly, the KEYNOTE-164 clinical trial has demonstrated durable anti-tumour activity of pembrolizumab in colorectal cancer patients, particularly those with high microsatellite instability (MSI) [31]. We hypothesise that pembrolizumab may have exerted some level of control on the colorectal tumour. However, further investigation is needed, particularly identifying the MSI status of the patient, to determine if he may have benefited from pembrolizumab treatment. The variability on detection of ctDNA across multiple cancers and tumour locations, also remains a topic of investigation in the field of liquid biopsy research.

In conclusion, ongoing close surveillance of melanoma patients who achieved complete response to BRAF inhibition and/or immune-checkpoint inhibitors is paramount to monitor potential recurrent disease. Emergent of new malignant lesions in this population should be regarded as a metastasis only after detailed evaluation, including a biopsy where feasible; otherwise there is a possibility of missing a secondary malignancy. Plasma ctDNA may aid in clarifying disease status of patients with metachronous cancer.

## Data Availability

All data analyzed for this case report has been presented within the manuscript. Data sharing is not applicable to this article as no datasets were generated or analysed during the current study.

## Abbreviations

AJCC: American joint committee on cancer
B: Bevacizumab
CEA: Carcinoembryonic antigen
ctDNA: Circulating tumour DNA
CTLA-4: Cytotoxic T-lymphocyte antigen 4
ddPCR: Droplet digital polymerase chain reaction
FDG-PET/CT: Fluorine 18 fluorodeoxyglucose- Positron emission tomography/ Computed tomography
FOLFOX: Folinic acid, fluorouracil and oxaliplatin chemotherapy
PD- 1: Programmed death antigen 1
RECIST: Response evaluation criteria in solid tumours
